# Rapid Accumulation of Total Lipid in* Rhizoclonium africanum* Kutzing as Biodiesel Feedstock under Nutrient Limitations and the Associated Changes at Cellular Level

**DOI:** 10.1155/2015/275035

**Published:** 2015-12-31

**Authors:** Gour Gopal Satpati, Sanjit Kanjilal, Rachapudi Badari Narayana Prasad, Ruma Pal

**Affiliations:** ^1^Phycology Laboratory, Department of Botany, University of Calcutta, 35 Ballygunge Circular Road, Kolkata, West Bengal 700019, India; ^2^Lipids Science and Technology, Council of Scientific and Industrial Research-Indian Institute of Chemical Technology, Hyderabad, Andhra Pradesh 500007, India

## Abstract

Increase of total lipid and the proportion of the favorable fatty acids in marine green filamentous macroalga* Rhizoclonium africanum* (Chlorophyceae) was studied under nitrate and phosphate limitations. These stresses were given by both eliminating and doubling the required amounts of nitrate and phosphate salts in the growth media. A significant twofold increase in total lipid (193.03 mg/g) was achieved in cells in absence of nitrate in the culture medium, followed by phosphate limitation (142.65 mg/g). The intracellular accumulation of neutral lipids was observed by fluorescence microscopy. The scanning electron microscopic study showed the major structural changes under nutrient starvation. Fourier transform infrared spectroscopy (FTIR) revealed the presence of ester (C-O-C stretching), ketone (C-C stretching), carboxylic acid (O-H bending), phosphine (P-H stretching), aromatic (C-H stretching and bending), and alcohol (O-H stretching and bending) groups in the treated cells indicating the high accumulation of lipid hydrocarbons in the treated cells. Elevated levels of fatty acids favorable for biodiesel production, that is, C_16:0_, C_16:1_, C_18:1_, and C_20:1_, were identified under nitrate- and phosphate-deficient conditions. This study shows that the manipulation of cultural conditions could affect the biosynthetic pathways leading to increased lipid production while increasing the proportion of fatty acids suitable for biodiesel production.

## 1. Introduction

Biofuels are biodegradable, nontoxic, carbon neutral fuels and are categorized into primary and secondary fuels. The algal biomass can be directly converted to biodiesel, bioethanol, and other sustainable products. Technologically, secondary biofuels are grouped into first-, second-, and third-generation biofuels on the basis of production strategy of raw materials [1]. Biodiesel production from renewable sources is widely considered to be one of the most sustainable alternatives to fossil fuels and is a viable means to combat the environmental impacts of fossil fuels on global warming [1–4].

Algae are photosynthetic, autotrophic micro- and macroorganisms ranging from single cell to multicellular forms. They can capture atmospheric CO_2_ and fix it into organic biomass which can be converted into energy carriers such as biodiesel [1, 5]. Microalgae could produce considerable amounts of lipids (up to 70–80% dry cell weight) [6, 7]. Microalgal taxa like* Botryococcus*,* Chlamydomonas*,* Chlorella*,* Dunaliella*,* Euglena*,* Nannochloropsis*,* Scenedesmus*,* Neochloris*, and so forth have already been identified as good sources for biodiesel production [1, 8]. But the main constraint of microalgal biomass production for biodiesel generation is the economic aspects. Macroalgal mat is one of the alternative livestock for sustainable biodiesel production in a cost-effective way. Only a few reports are available on biodiesel production from macroalgae or seaweeds [9–14]. It has been previously reported that some macroalgal species contain only very small quantities of total lipid as percentage of dry cell weight [10, 11, 15]. For instance, the total lipid percentages in* Spirogyra orientalis*,* Cladophora crystallina*, and* Chaetomorpha gracilis* were reported as 21 ± 2.5%, 23 ± 1.8%, and 16 ± 0.5%, respectively [11].* Rhizoclonium africanum*, a marine filamentous epiphytic macroalga, is found in association with mangrove plants. Filaments are stiff, entangled, and branched. Branches held out at right angles with the main axis. Cells are cylindrical and swollen with numerous rhizoidal branches [16–18].

Lipids in eukaryotic photosynthetic organisms function as a structural component of cell membranes that modulate cellular activity and serve as energy storage compounds [19]. The synthesis of neutral lipids in the form of triacylglycerol (TAG) within lipid body organelles is enhanced in response to different environmental stresses such as high light intensity or nutrient deprivation [20, 21]. Trigering enhanced synthesis of neutral lipids in green algae under stress conditions for biodiesel production has been previously reported [7]. In fact, the cells begin to accumulate oil in the form of cytoplasmic lipid bodies, specifically in the form of TAG [20, 22, 23]. High TAG accumulation in marine microalga* Dunaliella* cells under salt stress was studied in detail [22]. Some dinoflagellates also accumulate large quantities of TAG during the stationary phase of their growth period [24].

Some previous studies on lipid accumulation under nutrient stress conditions such as nitrogen starvation, phosphorous starvation, urea limitation, and iron supplementation were done in detail [22, 25–30]. The 2–4-fold increase in lipid content has been achieved in N-deficient freshwater using marine microalgae such as* Chlorella* and* Nannochloropsis* [25, 28]. The changes in lipid content under nitrogen deprivation were also observed in* Chlamydomonas reinhardtii* species [31, 32]. Nitrogen and phosphorous limitations were found to affect chlorophyll fluorescence of two macroalgae:* Ulva lactuca* and* Lobophora variegata* [14]. Nutrient uptake also played an important role in growth physiology of* Ulva intestinalis*,* Bifucaria bifurcata*, and* Nemalion helminthoides* [13]. The growth and biochemical changes of a red alga* Gracilaria tenuistipitata* var.* liui* and a green alga* Ulva pertusa* were also studied under nitrogen enrichment and starvation [9].

Despite all the efforts made to date, the effects of phosphate (PO_4_^−^) and nitrate (NO_3_^−^) starvation on production of monounsaturated (MUFA) and saturated fatty acids (SFA) and related parameters in macroalgae have not been extensively studied yet. In this study, our aim was to determine and compare the effects of such abiotic stresses on lipid production in* R. africanum*. Lipid peroxidation assay, FTIR, and fluorescent microscopy were also conducted to determine the increasing level of lipid accumulation in the stress-exposed cells.

## 2. Material and Methods

### 2.1. Culture Establishment in Unialgal Condition and Biomass Yield

*R. africanum* (CUH/Al/MW-57) was isolated from the coastal zone of Sundarbans and cultivated in a modified Bold Basal Medium (BBM) [33]. The composition of the BBM medium was manipulated based on two parameters: absence and presence of nutrients. The double doses of nitrate (DDN) (0.50 g/L) and phosphate (DDP) (0.15 g/L K_2_HPO_4_ and 0.35 g/L KH_2_PO_4_) were added in one set of experiments while, in the other set, biomass was exposed to the absence of nitrate (AN) and phosphate (AP). Other micro- and macronutrients were used in normal concentrations. The alga was grown at 20°C temperature and was exposed to 16 : 8 light-dark cycle with 135 rpm agitation in Eyela horizontal shaker-incubator. Biomass yield (g/L) in terms of dry cell weight (dcw) was measured gravimetrically [34].

### 2.2. Scanning Electron Microscopy (SEM)

SEM images were obtained using a Carl Zeiss EVO 18 (EDS 8100) microscope equipped with a Zeiss Inca Penta FETX 3 (Oxford Instruments). The sample material was washed with phosphate buffer saline (PBS) for 2-3 times and dried at room temperature. After complete drying, the samples were placed on a carbon tape and were coated by gold in Quorum (Q 150 TES). The photographs were taken at different magnification.

### 2.3. Estimation of Total Chlorophyll, Carbohydrate, and Protein

The growth performances under different stress conditions were studied by chlorophyll estimation. Total chlorophyll was estimated by the protocol described by Arnon [35]. Total carbohydrate content under nitrate and phosphate stress was studied by anthrone reagent [36]. Estimation of total protein was conducted by Lowry method [37].

### 2.4. Lipid Peroxidation Assay

Algal biomass at log exponential phase was collected and dried properly. About 0.5 g dried biomass was homogenized with 1 mL of 0.1% Trichloroacetic acid (TCA). The homogenate was centrifuged at 12,000 rpm for 15 min. About 500 *μ*L of supernatant was taken and mixed with 1 mL of 0.5% 2-thiobarbituric acid (TBA). The mixture was boiled for 30 minutes in water bath at 95°C. The mixture was then cooled in ice and centrifuged at 10,000 rpm for 15 min. The optical density was measured at 532 nm and 600 nm.

### 2.5. Gravimetric Determination of Total Lipid

About 0.356 g of dried algal biomass was ground and mixed with 2 mL of chloroform, 2 mL of methanol, and 1 mL of 5% NaCl solution [38]. The mixture was vortexed for 2-3 minutes and centrifuged at 10,000 rpm for 5 min at 20°C. Chloroform layer was collected carefully. The same process was repeated 2-3 times and the collected chloroform samples were pooled and evaporated using a rotary evaporator at room temperature. The lipid residue was dried in an oven at 60°C and weighed in order to obtain the lipid content (%) in dry biomass.

### 2.6. Fatty Acid Methyl Ester (FAME) Production by Transesterification

FAMEs were produced by the transesterification method. The lipid samples after extraction were taken into a 10 mL screw-cap glass tube (BOROSIL, Mumbai, India) in which the transesterification reagents methanolic hydrochloric acid (1 : 4 v/v) was added. The tube was kept in a glass beaker containing some double distilled water and heated in a hot air oven at 70°C for 6–8 h. The solution was allowed to cool and centrifuged at 10,000 rpm for 10 min to avoid particulate matters. The FAME extract was then transferred to GC-MS autosample vials for analysis.

### 2.7. Gas Chromatography-Mass Spectrometry (GC-MS)

The FAME was subjected to GC-MS detection performed with Agilent 6890N Gas Chromatograph connected to Agilent 5973 Mass Selective Detector at 70 eV (*m*/*z* 50–550; source at 230°C and quadruple at 150°C) in the electron impact mode with a HP-5 ms capillary column (30 m × 0.25 mm i.d. × 0.25 *μ*m film thickness). The oven temperature was programmed for 2 min at 160°C and raised to 300°C at 5°C/min and maintained for 20 min at 300°C. The carrier gas, helium, was used at a flow rate of 1.0 mL/min. The inlet temp was maintained at 300°C, and the split ratio was 50 : 1. Structural assignments were based on interpretation of mass spectrometric fragmentation and confirmed by comparison of retention times as well as fragmentation patterns of authentic compounds. GC analysis was performed on a HP 6850 Series gas chromatograph equipped with a FID detector and DB-225 capillary column (30 m × 0.25 mm I'd. × 0.25 *μ*m film thicknesses). The injector and detector temperatures were maintained at 300 and 325°C, respectively. The oven temperature was programmed for 2 min at 160°C and raised to 300°C at 5°C/min and maintained for 20 min at 300°C. The carrier gas, nitrogen, was used at a flow rate of 1.5 mL/min. The injection volume was 1 *μ*L, with a split ratio of 50 : 1. The identification of individual fatty acids was done on the basis of retention time.

### 2.8. Fluorescent Microscopic Study of Neutral Lipid

The accumulation of neutral lipids in cell cytoplasm was observed by an Olympus U-RFL-T (Model BX-51) fluorescent microscope using red filter. The algal cells were stained with Nile red (0.1 mg in 1 mL acetone) and incubated for 10 min in dark. The cells were washed 2-3 times with PBS at pH 7.4 and slides were prepared with 10% glycerine (v/v) solution. The photographs were taken using an Olympus cool snap cf color/OL microscope at 10x and 40x magnifications.

### 2.9. Fourier Transform Infrared Spectroscopy (FT-IR) for Determination of Functional Groups

The algal biomass in log phase was collected and washed 2-3 times with double distilled water. After washing, the biomass was blotted and dried in a hot air oven at 70°C to achieve complete dryness. About 0.1 mg of algal powder was mixed with 0.1 mg of KBr and the functional groups were analyzed using a Perkin Elmer FTIR (Perkin Elmer, USA).

### 2.10. Statistical Analysis

Statistical analysis was performed using a linear regression plot by Microsoft Office Excel 2007. The relationship between lipid and the other bioactive compounds was studied by linear regression plot. One-way ANOVA analysis was done to perform statistical relationships of all bioactive compounds with different experimental conditions. Statistical significance was assessed at the level of *P* = 0.05 and *P* = 0.01.

## 3. Results and Discussions

### 3.1. Changes in Cell Morphology

**Figure 1:**
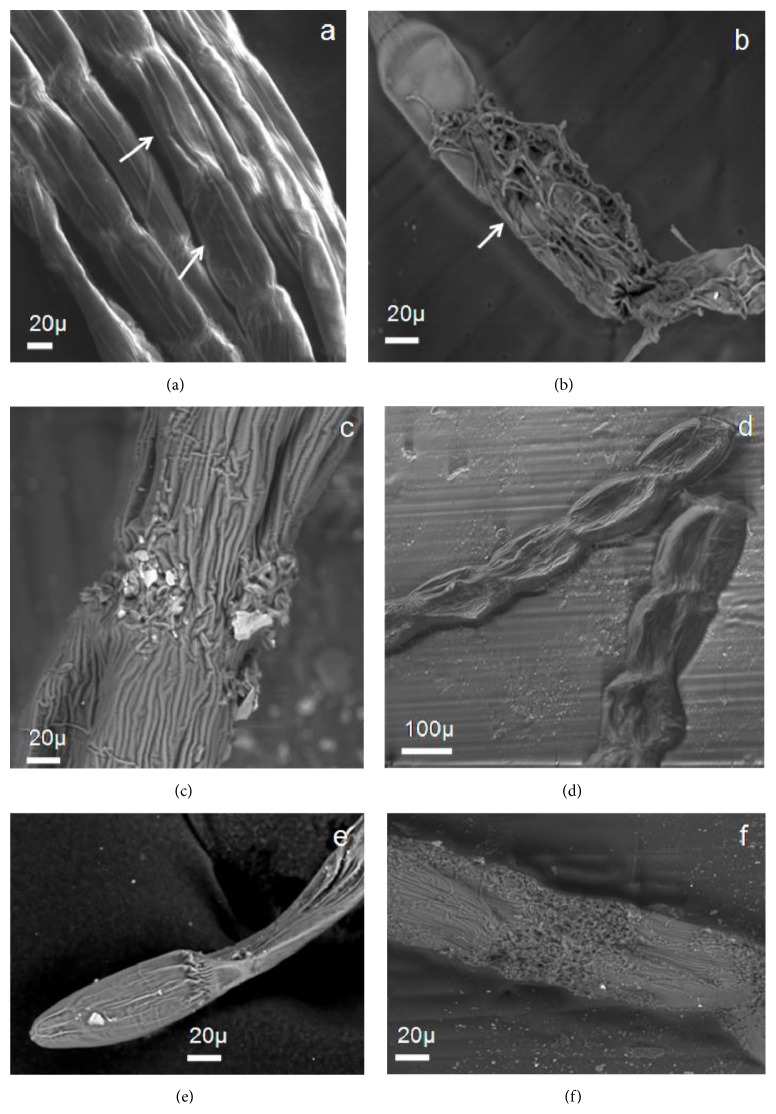
Showing SEM micrographs of* R. africanum* under nitrate and phosphate stress. (a) Untreated intact cell (×1.0 KX). (b) Disintegrated cell wall polysaccharides of AN treated cell (×1.0 KX). (c) Cell with degraded cellulose macrofibrils in AP condition (×1.0 KX). (d) Cells become swollen and rectangular to oval in DDP media (×256 X). (e) A terminal cell with folded margins in DDP treated condition (×1.0 KX). (f) Cross wall with greater folding of cellulosic macrofibrils in DDN treated cell (×500 X).

SEM micrographs showed intact cell walls of the control cells whereas disintegration of cell wall polysaccharides was observed in DDN treated cells (Figures 1(a) and 1(b)). In AN treated cell, cell surface was found to be ruptured and disorganized (Figure 1(c)). Different patterns of cell morphology were observed under the DDP treated condition (Figure 1(d)). Terminal cells become more elongated with folded margins under DDP treated condition (Figure 1(e)) but AP led to disorganization of cross walls between cells (Figure 1(f)). In our previous report, similar observations were recorded in filamentous green alga* Spirogyra punctulata* under nitrate, phosphate, and sodium chloride stress [39]. Degradation of cell wall and formation of abnormal chloroplasts were observed in nutrient-deficient conditions. In this study, morphological changes were observed under nitrate and phosphate deficiency and abundance.

### 3.2. Growth Characteristics

**Figure 2:**
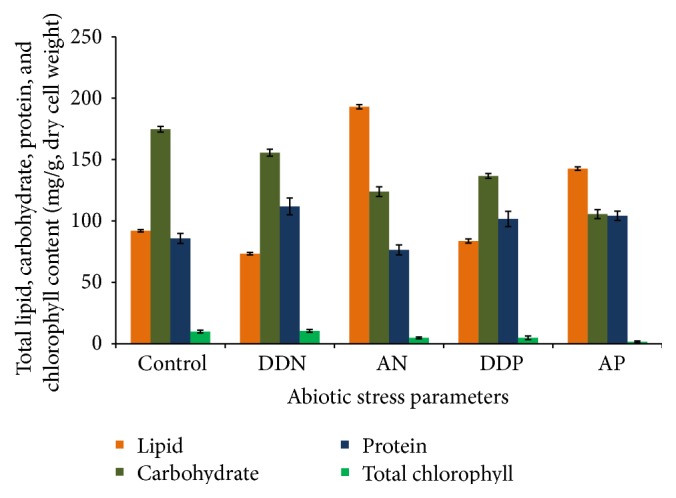
Bar graphs showing total chlorophyll, carbohydrate, protein, and lipid content (mg/g) of both control and treated biomass (DDN, AN, DDP, and AP) in dry weight basis. *P* = probability value (significant level); *P* ≤ 0.05.

The growth patterns of the alga under both control and treatment conditions were determined in terms of chlorophyll content (mg/g) and biomass yield (g/L). Under DDN condition, the growth of the alga was maximal as designated by high chlorophyll content (10.55 mg/g) and dry biomass weight (3.4 g/L), compared to the untreated cells (Figure 2). A sharp decline in total chlorophyll content was observed in DDP (4.905 mg/g), AN (4.874 mg/g), and AP (1.681 mg/g) treated cells, respectively (Figure 2). In different growth period (early, mid-, and late growth), chlorophyll a was measured at low, intermediate, and high nitrogen concentrations using two microalgae:* Chlamydomonas reinhardtii* and* Scenedesmus subspicatus* [2]. In this study, total chlorophyll content was determined and found to be high in nutrient sufficient condition and less in nutrient depleted condition.

**Figure 3:**
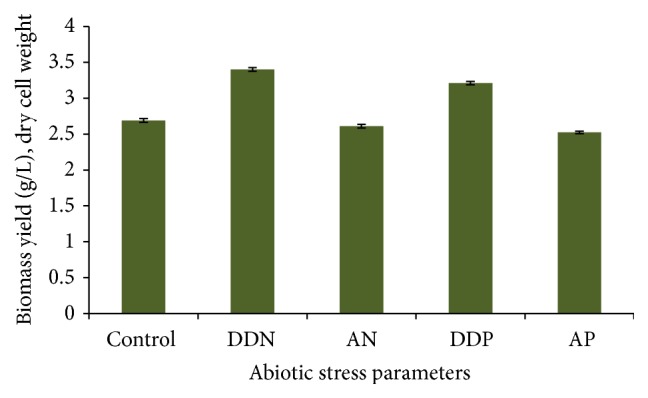
Bar graphs showing biomass yield (g/L) of* R*.* africanum* in log phase (14 days) under different nutrient limited conditions (DDN, AN, DDP, and AP). *P* = probability value (significant level); *P* ≤ 0.05.

The cell growth in terms of biomass concentration (mg/L) was studied in* Monoraphidium* sp. under different concentration of nitrogen in the culture [40]. In microalgae, several studies have been done on growth and biomass yield under nitrate and phosphate stress [34, 41]. Here, we determined growth of* R. africanum* in terms of total chlorophyll content (mg/g) and biomass yield (g/L, dry cell weight). The biomass yield under DDP, AN, and AP stresses was recorded as 3.21, 2.61, and 2.52 g/L, respectively (Figure 3). The growth in terms of total chlorophyll and biomass yield was found to be significant in all experimental conditions (*P* < 0.001) except AN and AP (Figures 2 and 3).

It has been reported that, in macroalgae, chlorophyll and phycoerythrin contents were enhanced significantly after 10 d of incubation with increasing concentration of ammonium (NH_4_^+^) [9]. The biomass growth of other macroalgal taxa including* Bifurcaria bifurcata*,* U. intestinalis*, and* Nemalion helminthoides* was also studied under different concentrations of nitrogen and phosphorus [13].

### 3.3. Changes of Total Carbohydrate and Total Protein

The high carbohydrate content was measured in the untreated cells (174.66 mg/g) followed by DDN (155.62 mg/g) and DDP (136.64 mg/g) treatments (Figure 2). Most of the studies have been done on the production of total carbohydrate and protein content of marine macroalgae [10, 42, 43]. The major findings of our current study were mainly based on the changes of cellular carbohydrate and protein content in relation to lipid and fatty acids. The nitrate and phosphate depletion resulted in a sharp decrease in carbohydrate content with time (Figure 2). There was a significant reduction in carbohydrate content of all the treated cells (*P* < 0.001) (Figure 2). The protein level was rapidly increased by the DDN (111.8867 mg/g), followed by AP (104.23 mg/g) and DDP (101.6833 mg/g), while the AN led to declined total protein content in the cells (76.48 mg/g) (Figure 2). The interaction of protein with nitrate and phosphate concentration was significant in DDN, AP, and DDP treated cells (*P* < 0.05) except for the AP treated cells (*P* > 0.05) (Figure 2). It has been observed that, in* Gracilaria* and* Ulva*, the total protein content was increased significantly after 10 d of ammonium enrichment [9]. Carbohydrate and protein are among the most important components involved in metabolism for they supply energy for growth and cellular differentiation. Seasonal variation of total carbohydrate and protein content of the marine macroalgae* Enteromorpha intestinalis*,* Ulva lactuca*, and* Catenella repens* from coastal West Bengal was studied by Banerjee et al. [44]. It has been previously reported that carbohydrate accumulation is inversely proportional to the lipid production, since the lipid precursor glycerol-3-phosphate is produced by glucose metabolism [45, 46].

### 3.4. Changes of Total Lipid

Twofold increase in total lipid content (193.03 mg/g) was found in AN treated biomass of* Rhizoclonium* (Figure 2). Moreover, the AP resulted in lipid productivity up to 142.65 mg/g, which was 1.5-fold more than that of the untreated cells (92.07 mg/g) (Figure 2). The results obtained indicated that both phosphate and nitrate stress induced lipid biosynthesis in green macroalgal filament (DDN, AN, DDP, and AP treated cells). The results showed a significant increase in AN and AP treated condition and a significant decrease in DDN and DDP treated condition (*P* < 0.05). Similar observations were made in several studies on various microalgae [47–49]. The lipid content of* Spirogyra* and* Chara* was reported by Trifa et al. [50]. The lipid classes of macrophytic algae from different groups, Phaeophyta, Rhodophyta, and Chlorophyta, were determined at various seasons [51]. In this study,* Ulva lobata* of Chlorophyta contained the highest amount of lipids (20–29 mg/g dry biomass). Tran et al. had previously reported the effects of different nitrogen source in oil accumulation of a microalga,* Botryococcus braunii* [52]. They used (NH_4_)_2_CO_3_, urea, and NaNO_3_ as nitrogen source and fed the alga with different concentrations to study lipid accumulation.

Lipid productivity associated with biomass yield is an important criterion of oil-producing capacity. In this study, the highest lipid content was obtained under AN followed by AP. Widjaja reported that the lipid content of* Chlorella vulgaris* increased to 42% under nitrogen deficiency condition and the lipid productivity was 13 mg/L/d [53]. In a recent study, cellular biochemical responses have been analysed in an oleaginous microalga,* Chlorella vulgaris,* under different concentration of urea as nitrogen source [54].

Nitrogen and phosphorous are most important elements contributing to algal cells; its deprivation significantly changed the physiological and biochemical parameters [55].

### 3.5. Lipid Peroxidase Activity by Determination of Malonaldehyde (MDA)

**Figure 4:**
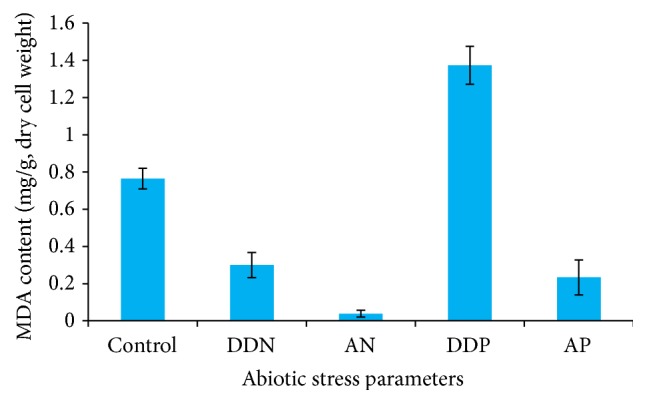
Bar graphs showing lipid peroxidase activity (MDA content) of both control and treated biomass (DDN, AN, DDP, and AP) of* R*.* africanum*. *P* = probability value (significant level); *P* ≤ 0.05.

The lipid peroxidation assay revealed a twofold increase (1.373 mg/g) in the production of malonaldehyde (MDA) content in DDP treated cells. Less peroxidation was observed in AN (0.03 mg/g) and AP (0.23 mg/g) treated cells (Figure 4). The less peroxidation indicated the high lipid accumulation caused by nutrient starvation. The significant relationship of the MDA with nutrient was obtained in DDP (*P* < 0.001) except for DDN, AN, and AP (*P* > 0.001).

Nutrient limitation affects lipid metabolism, which includes qualitative and quantitative alterations of lipid classes, inhibition of biosynthetic pathways, and production of unsaturated fatty acids due to nutrient enhanced peroxidation [20, 41, 56, 57]. In the present investigation, it has been shown that MDA content was found to be very low (0.03 mg/g), suggesting high cellular lipid accumulation in AN treated cells.

### 3.6. Fatty Acid Profiling

The fatty acid profile of* R*.* africanum* was comprehensively identified and quantified by GC-MS indicating high amounts of SFA and MUFA under stress conditions compared to the control (Table 1). The polyunsaturated fatty acid (PUFA) production rapidly declined under stress condition. An increase in saturated and monounsaturated fatty acids and decrease in polyunsaturated fatty acids have also been obtained in phosphate limited microalgae [20, 41, 58, 59]. The SFAs were found as C_12:0_, C_14:0_, C_16:0_, C_16:2_, C_17:0_, C_18:0_, C_22:0_, and C_24:0_. Among those, C_16:0_ made up the highest proportion under both control (30.2%) and stress conditions (40.9%). The AN treatment resulted in enhanced biosynthesis of MUFAs after a 14 d exposure, that is, C_16:1_, C_18:1_, and C_20:1_ (13.1, 29.2, and 1.7% in the treated cells compared to 9.4, 20, and 0% in the control, resp.) (Table 1). The fatty acid profile of* Ulva rigida* showed dominance of both saturated and unsaturated fatty acids [10]. The fatty acid profile of macrophytic algae* Egregia menziesii* (Phaeophyta),* Chondracanthus canaliculatus* (Rhodophyta), and* Ulva lobata* (Chlorophyta) showed dominant fatty acids of C_16:0_ and other fatty acid classes were found as C_14:0_, C_18:1_, C_20:4_, and C_20:5_ [51]. They also reported that the C_22_ PUFA were unique to* U*.* lobata*. The fatty acid analysis of* Ulva reticulata* showed dominance of C_16:0_ and C_14:0_ (50.76% and 11.77%) under control conditions [15]. The present research group reported fatty acid profiles of 21 micro- and macroalgal taxa from Indian Sundarbans [11]. In this study, the synthesis of high amount of MUFA and SFA in the cells indicated the high potential of this alga for biodiesel application. The C_16:1_, C_18:1_, and C_20:1_ were the major MUFA which synthesized within the cell under nutrient stress conditions. The absence of C_20:4_ and C_20:5_ in the treated cells was also observed. MUFA generally increases the biodiesel quality in terms of lubricity and the cetane number which are most applicable for biodiesel production [60]. Different classes of fatty acids, namely, MUFA and PUFA, of 100 macroalgal species were determined in context to their chemotaxonomic and nutritional perspectives [61]. The highest fatty acid content of brown seaweed,* Spatoglossum macrodontum* (57.4 mg g^−1^ dry weight), suggested that this taxon can be used for oil-based biodiesel [12]. The saturated and unsaturated fatty acids of 6 Arctic and 14 Antarctic macroalgae species from different groups, namely, Rhodophyta, Phaeophyta, and Chlorophyta, from Antarctic Peninsula were investigated [62]. These macroalgal species were cultivated in nutrient-enriched seawater at low temperatures (0–5°C) and natural light irradiance. In this study, they have found that the principal saturated fatty acid was C_16:0_. A high percentage (11.1%) of uncommon MUFA, C_16:1_ (n-5), was found in* Desmarestia muelleri* sporophytes were also investigated in this study. The PUFA of 17 macroalgal species from three different phyla (Chlorophyta, Rhodophyta, and Phaeophyta) were analyzed and major fatty acid classes were recorded as C_16_ and C_18_ [63].

The fatty acid composition of both micro- and macroalgae can vary both qualitatively and quantitatively with their physiological and biological status and culture conditions. The properties of biodiesel are mainly determined by its fatty acid esters [64]. The GC-MS study revealed that the biodiesel produced from* Rhizoclonium africanum* grown under the presence or absence of nitrate and phosphate was predominated with both saturated and monounsaturated fatty acid components, which is desired for good quality biodiesel. Interestingly, production of PUFA was subsequently decreased in the biodiesel produced under these stress conditions. The study of* Micractinium reisseri* showed major proportions of *α*-linolenic, linoleic, palmitic, and stearic acid [65]. Similar studies were performed by Lee et al. and Choi et al. [66, 67].

### 3.7. Observation of Cytosolic Neutral Lipids by Fluorescent Microscopy

**Figure 5:**
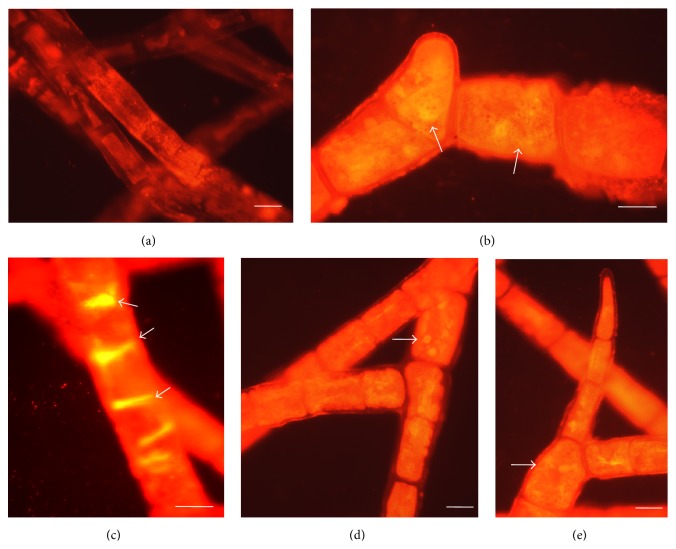
Showing fluorescent images of* R*.* africanum*. (a) Untreated cells with red chlorophyll autofluorescence. (b) Accumulation of neutral lipid in rhizoidal branch after DDN in the culture. (c) Bright yellow fluorescence due to the accumulation of more neutral lipid in AN treated culture. (d) Rhizoidal branch with nonpolar lipid droplets (yellow droplets) in DDP added culture. (e) Accumulation of less nonpolar lipid in the rhizoidal branch under AP.

Accumulations of cytosolic neutral (nonpolar) lipids in treated algal cells were studied by fluorescent microscopy (Figure 5). Cells exposed to AN showed bright yellow fluorescence (Figure 5(c)) of neutral lipids in cytosol compared to the untreated cell. Similar studies were reported in a microalga,* Nannochloropsis oculata,* when nitrogen limitations to the cells were abrupt and progressive mode [46]. It is well established that microalgae usually accumulate more lipids under abiotic and biotic stress conditions especially nutrient deficiency. For example, nitrogen starvation leads to higher lipid contents in many microalgal species [7, 34, 46]. Phosphorous deficiency simultaneously induces lipid accumulation in a variety of microalgal species [58]. In our present study, the accumulation of intracellular lipid bodies in the cell cytoplasm of* R. africanum* was investigated.

The untreated cell showed bright red autofluorescence for the presence of chlorophyll a and chlorophyll b (Figure 5(a)). Light yellow fluorescence of nonpolar lipids was also studied in DDN, DDP, and AP treated cells (Figures 5(a), 5(d), and 5(e)). The confocal images of* Chlorella ellipsoidea* and* Chlorococcum infusionum* showed an enhanced accumulation of neutral lipids in form of droplets under nitrate starvation [34]. The macroalga* Rhizoclonium africanum* showed high accumulation of neutral lipids under nutrient starvation (Figure 5). Similar studies were performed using macroalgae and seagrass and a characteristic change was observed under nutrient limitation [14]. They used nutrient induced fluorescence technique (NIFT) to detect fluorescence intensity among* Ulva lactuca*,* Lobophora variegata*, and* Thalassia testudinum*.

It has been suggested that an increase in total cellular lipid was due to an increase in neutral lipids [68]. More scientifically, it can be stated that nitrate and phosphate deficiency leads to an increase in production of triacylglycerol in algae [34, 68].

### 3.8. Study of Functional Groups by FTIR Spectroscopy

**Figure 6:**
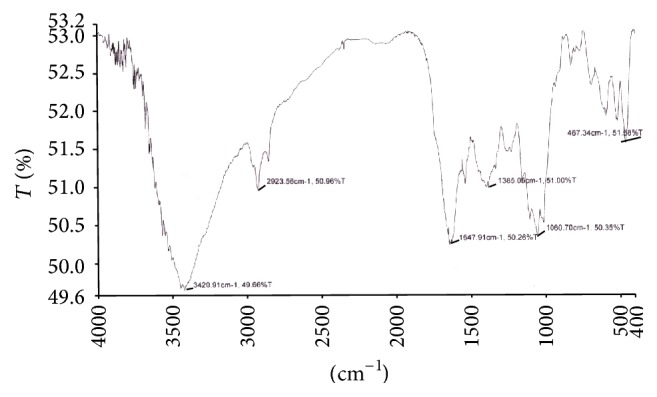
FTIR spectra of control biomass showing different functional groups. The “*x*” axis of the spectra denotes wavenumber (cm^−1^) and “*y*” axis denotes transmittance (% *T*).

**Figure 7:**
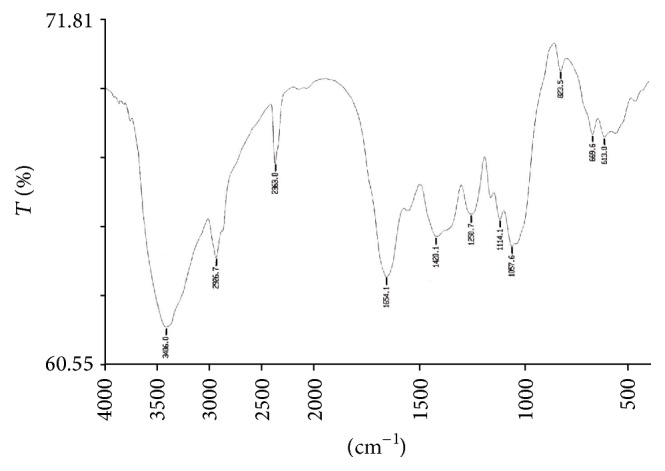
FTIR spectra of +NO_3_ (DDN) treated biomass showing different functional groups. The “*x*” axis of the spectra denotes wavenumber (cm^−1^) and “*y*” axis denotes transmittance (% *T*).

**Figure 8:**
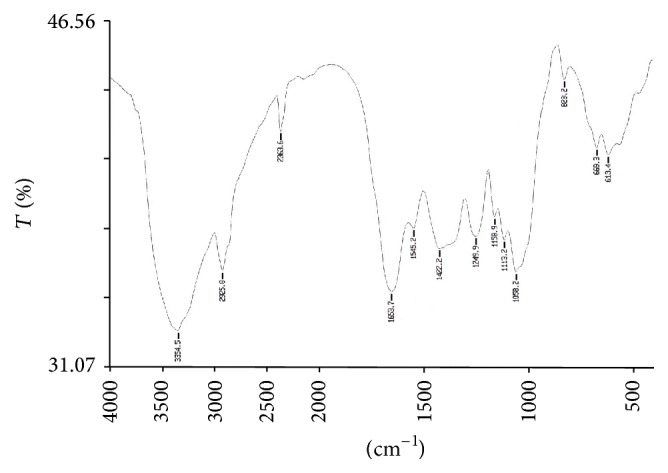
FTIR spectra of −NO_3_ (AN) treated biomass showing different functional groups. The “*x*” axis of the spectra denotes wavenumber (cm^−1^) and “*y*” axis denotes transmittance (% *T*).

**Figure 9:**
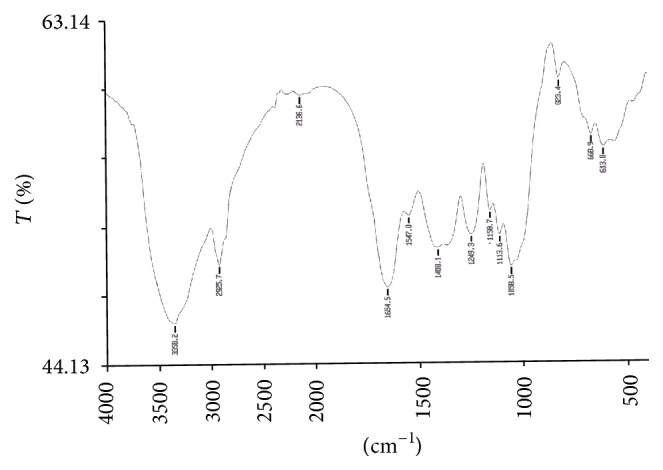
FTIR spectra of +PO_4_ (DDP) treated biomass showing different functional groups. The “*x*” axis of the spectra denotes wavenumber (cm^−1^) and “*y*” axis denotes transmittance (% *T*).

**Figure 10:**
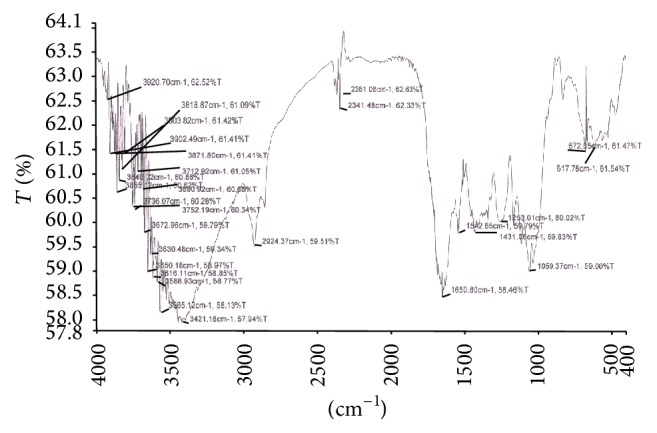
FTIR spectra of −PO_4_ (AP) treated biomass showing different functional groups. The “*x*” axis of the spectra denotes wavenumber (cm^−1^) and “*y*” axis denotes transmittance (% *T*).

The FTIR spectroscopy is a most sophisticated method for whole organism analysis using intact cells, which involves the measurement of infrared absorption in relation to a range of molecular vibrational modes [2]. Specific functional groups can be identified by their absorption bands. A few reports were begun to demonstrate the potential of FTIR as a tool to identify changes in cellular components, including lipids, in response to nutritional stress [2, 69, 70]. In this study, the FTIR spectra of the control biomass of* R. africanum* were compared with those under nutrient-deficient conditions (Figures 6–10). The spectra of both nitrate and phosphate treated biomass indicated the presence of ester, ketone, carboxylic acid, phosphine, aromatic, and alcohol functional groups (Figure 7). Display of bond C-O-C stretch ester in the region of 1249.9, 1249.3, and 1253.01 cm^−1^ for lipids was observed in nitrate- and phosphate-deficient conditions. The C-C stretching for lipid ester was obtained in the region 1250.7 cm^−1^. The peaks appearing in the region of 1114.1 (DDN); 1113.2, 1158.9 (AN); and 1113.6, 1158.7 cm^−1^ (DDP) might be attributed to C-C stretch of ketone. Presence of ketone and ester in treated biomass indicated the synthesis of lipids in the cells under nutrient starvations. The peaks appearing in the region of 3354.5 cm^−1^ (in AN treated sample) (Figure 8) and 3650.18–3920.70 cm^−1^ indicated the presence of high degree of stretching of O-H alcoholic group whereas the region of 3616.11 and 3630.48 cm^−1^ signified bending of O-H for alcohol in the AP treated biomass (Figure 10). The O-H bending for carboxylic acid appeared in the region of 1420.1, 1422.2, 1408.1, and 1431.08 cm^−1^ in all treated samples. A single peak of O-H stretching for alcohol in biomass of DDN medium was obtained in the region of 3406 cm^−1^ (Figure 7). An analysis of the infrared (IR) spectrum showed the existence of the absorption bands characterized by C=O, C-O-C, C-H, CO_2_, and H_2_O in the range of 900–2875 cm^−1^ [71]. This study has been done with an isolated indigenous green microalga,* Chlorella vulgaris*. In our investigation, we have found a wide range of functional groups of different biomolecules under nutrient stress conditions.

The presence of P-H stretching for phosphine group was obtained in the region of 2363, 2363.6, 2361.06, and 2341.48 cm^−1^ in DDN, AN, and AP treated biomass, respectively. The C=O and N-H stretching and bending for amide group of protein were obtained in both control and treated biomass in the region of 3421.18, 3420.91, 1654.1, 1653.7, 1547, 1545.2, and 1542.65 cm^−1^, respectively. The peaks at 1060.7, 1057.6, 1058.2, 1058.5, and 1059.37 cm^−1^ were caused due to the C-N amine stretching of polypeptides in all the treated and control biomass. The peaks of N-H stretch of amine group were obtained in the region of 3358.2, 3588.93, and 3565.12 cm^−1^ in phosphate treated biomass (Figures 9 and 10). The peaks at 823.5, 823.2, and 823.4 cm^−1^ were caused by C-H bending of aromatic group in DDN, AN, and DDP treated biomass, respectively. The alkyl halide groups (C-I, C-Cl stretching) were found in control and AP treated biomass lying in the region of 467.34 and 672.05 cm^−1^. The C-H stretch for alkanes wwas obtained in the region of 2923.56, 2924.37, 2925.7, 2925.8, and 2926.7 cm^−1^, respectively, in all the treated and control biomass. The peaks at 1647.91, 1650.8, and 1654.5 cm^−1^ were caused by C=C stretching of alkenes in control, AP, and DDP treated biomass, respectively (Figures 9 and 10). The out-of-plane bending of C-H for alkenes was found in the region of 669.3 and 668.9 cm^−1^ in AN and DDP treated biomass only (Figures 8 and 9). Only one peak at 1385.06 cm^−1^ was obtained due to the C-H plane bending of alkenes group in control biomass (Figure 6). The C-H stretching and bending of alkynes were obtained in the region of 613, 613.4, 617.78, and 669.6 cm^−1^ after induction of all the nitrate and phosphate stress. The FTIR spectra of* Chlamydomonas reinhardtii* cells showed nine distinct absorption bands over the wavenumber range of 800–1900 cm^−1^ [2]. In the present investigation, multiple absorption bands were found in* R. africanum* under nitrate and phosphate starvation, resulting in ester, ketone, carboxylic acid, and alcohol groups. The FTIR spectroscopic analysis revealed the detection of C-O-C stretching for esters of lipid in the region of 1249.9 cm^−1^ (in AN), 1249.3 cm^−1^ (DDP), and 1253.01 cm^−1^ (AP), respectively.

The synthesis of aromatic compounds, phosphine, ketone, alcohols, and carboxylic acids in the treated cells was observed (Figures 6–10). It has been stated that the lipid and other bioactive compounds were changed significantly under nutrient stress. These functional groups were not synthesized in the untreated cells of the biomass. The infrared spectra for relative detection of triacylglycerol, oligosaccharides, and polysaccharides were studied under nitrogen and sulfur deprived conditions [72]. Using infrared spectroscopy, detection of lipid, protein, and carbohydrates in both untreated and treated microalgal cells under different abiotic conditions was also studied for several times [2]. But in the present study our aim was to demonstrate the accumulation of lipid and other bioactive molecules in a macroalga,* R. africanum*, using FTIR spectroscopy.

### 3.9. Statistical Analysis

**Figure 11:**
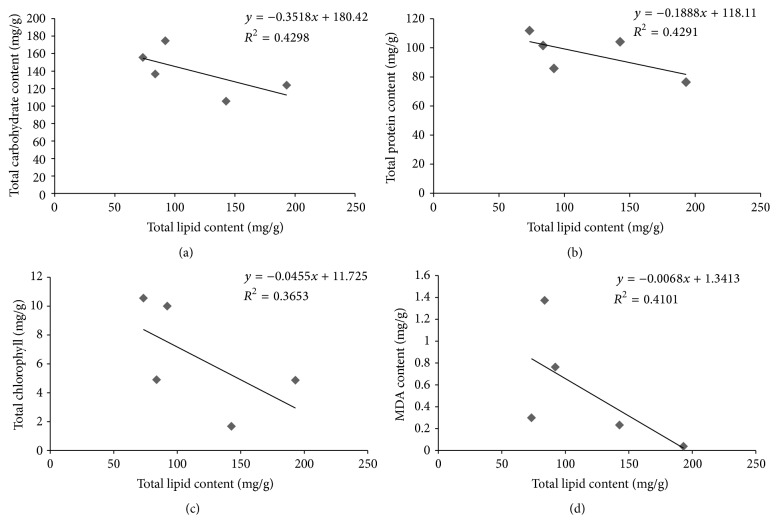
Showing linear regression plot of relationship between lipid and other bioactive compounds—chlorophyll, carbohydrate, protein, and lipid peroxidase (MDA).

The correlation coefficient was found to be insignificant when total lipid content was compared with total chlorophyll (*R* ^2^ = 0.365) (Figure 11(c)) and carbohydrate content (*R* ^2^ = 0.429) (Figure 11(a)). The correlation coefficient of total protein content and MDA content was also found to be insignificant (*R* ^2^ = 0.429 (Figure 11(b)) and *R* ^2^ = 0.410 (Figure 11(d))) in comparison with the total lipid content. A positive correlation (*R* ^2^ = 0.67) between the fatty acids and total lipid content was documented in the macroalgae* Spatoglossum macrodontum* and* Derbesia tenuissima* by Gosch et al. [12].

In the present study, two main factors like the presence and absence of nutrients were applied on the green macroalga* R. africanum* and it was reported that maximum lipid accumulation took place for AN treated cells. This result was supported by GC-MS (high SFA and MUFA) and fluorescent microscopy as well. Bright yellow fluorescence of neutral lipid in the form of triacylglycerol (TAG) was observed in cytosol of AN treated cell. In these cells, the protein decreased in general and such observation was also reported earlier by Reitan et al. [73]. The effects of nitrogen-deficient medium for a period of 7 to 17 days on* Chlorella vulgaris* were studied earlier by Widjaja [53]. They observed an increase in total lipid at the end of the 17 d culture period. In our observation, both AN and AP treated cells showed high lipid content due to the breakdown of starch into acetyl CoA. The accumulation of high carbon in AN and AP treated cells triggers the synthesis of intracellular lipid in* R. africanum*. The low carbohydrate content in both the AN and AP treated cells was associated with increase in cellular lipid accumulation. The degradation of cell wall polysaccharides (Figures 1(b), 1(c), and 1(f)) after 14 d exposure in AN and AP may suggest the breakdown of polysaccharides into monosaccharides. The SEM studies supported that degradation of cell wall polysaccharides under stress condition (Figure 1).

Increased Nile red fluorescence of neutral lipid and gravimetric yield of total cellular lipids clearly suggested that nitrate and phosphate deprivation stimulated lipid storage in* R. africanum*. It has been found that, under sufficient nutrients, carbohydrates and proteins are synthesized; however, in nutrient limited condition, cell division is arrested and greater amount of carbon is available for lipid storage [68].

In many algae, lipid synthesis has been stimulated by the depletion or removal of nitrate and phosphate from the culture media. In* R. africanum*, the 2-fold increase of lipid under the absence of nitrate and 1.5-fold increase under the absence of phosphate were investigated.

In our investigation, the growth rate of* R. africanum* was greatly reduced under the absence of nitrate and phosphate but did not completely cease. Simultaneously the double doses of nitrate and phosphate trigger growth of* R. africanum*. Similar observations were bottom in disparate microalgal species, yet not a well-known report is ready to be drawn in macroalgae. The growth of this alga was mainly stimulated by the rapid accumulation of carbohydrate and protein in the cells under elevated level of nitrate and phosphate in the culture. A very recent study has demonstrated significant increase and decrease of biomass in a microalga,* Nannochloropsis oculata*, under various concentration of nitrate in the culture [46].

To the best of our knowledge, this was the first time a macroalga was used for high lipid and other macromolecules production while the presence and absence of key nutrients in the culture were also investigated. In this study it was shown that while the protein content decreased, the lipid level increased under the AN condition. Less production of MDA in the AN and AP treated cells indicated low peroxidation of lipids leading to high lipid productivity.

The nutrient deficiency enhanced the production of chlorophyll content which might provide more alkaline pH, Mg^2+^, and NADPH to enhance ACCase activity. However, more lipid accumulation takes place under nutrient limitation than under nutrient saturation. It has been reported that the nitrogen limitation impaired the cellular abundance and activity of ACCase enzyme, but cell division almost ceased, resulting in the accumulation of lipid [74–77]. Therefore, it can be suggested from the present investigation that macroalgal biomass can also be exploited as lipid feedstock for biodiesel production. Hence the ability to physiologically manipulate the quality and quantity of lipid, fatty acids, protein, and carbohydrates in* R. africanum* would thus be significant for biodiesel and several different applications. However, more investigations are required to verify this process in large scale cultivation with regard to technical and economic aspects.

## 4. Conclusions

From the above study it can be concluded that both nitrate and phosphate starvation enhanced the lipid productivity as well as other cellular changes, namely, protein, carbohydrate, and so forth. The accumulation of cytosolic neutral lipid (from fluorescent microscopy) and increased MUFA and SFA in the alga supported this result. The maximum lipid productivity (in terms of mg/g) was observed in nitrate and phosphate depleted cells. The degradation of cell wall (from SEM study) indicated the conversion of carbohydrate to lipid and fatty acids. The above abiotic conditions can be successfully applied for large scale cultivation and processing of macroalgal biomass for production of biodiesel. The use of natural filamentous alga can be used as an alternative for third-generation biodiesel at a cost-effective way.

## Figures and Tables

**Table 1:** Showing fatty acid compositions (%) of both control and treated cells (DDN, AN, DDP, and AP) of *R*. *africanum* at log-exponential phase (14-day-old culture).

Fatty acids	Fatty acid compositions (%)				
	Control (14 days)	+NO_3_ (DDN) (14 days)	−NO_3_ (AN) (14 days)	+PO_4_ (DDP) (14 days)	−PO_4_ (AP) (14 days)
12:0	3.4	1.1	—	0.5	0.6
14:0	6.5	4.9	4	6.6	5.2
15:0	2.8	1.2	1.1	2.4	2
16:0	30.2	34.9	32.6	29.9	40.9
16:1	9.4	11.2	13.1	11.2	12.2
16:2	—	0.8	1	1.6	1.5
16:3	—	0.9	0.2	5.2	0.8
17:0	—	—	—	0.2	—
18:0	1.4	1.3	1.3	0.6	1.3
18:1	20	21.2	29.2	20.2	23.9
18:2	5.3	5.3	4.1	10.7	5.1
18:3 (GLA)	—	1.1	0.9	1.7	1.2
18:3 (ALA)	7.4	—	—	—	—
20:0	—	—	0.6	—	—
20:1	—	1.3	1.7	0.4	1.5
20:2	—	—	0.9	1.4	—
20:3	2.4	1.2	2	3.6	—
20:4	—	—	—	—	—
20:5	—	—	—	—	—
22:0	—	—	—	0.2	—
24:0	11.2	11.5	12.1	3.4	6.2
